# Association between the *NBS1 *E185Q polymorphism and cancer risk: a meta-analysis

**DOI:** 10.1186/1471-2407-9-124

**Published:** 2009-04-24

**Authors:** Meixia Lu, Jiachun Lu, Xiaobo Yang, Miao Yang, Hao Tan, Bai Yun, Luyuan Shi

**Affiliations:** 1 Department of Epidemiology and Statistics, School of Public Health, Tongji Medical College, Huazhong University of Science and Technology, Wuhan 430030, PR China; 2 Institute of Occupational Medicine and The MOE Key Lab of Environment and Health, School of Public Health, Tongji Medical College, Huazhong University of Science and Technology, Wuhan 430030, PR China; 3 The Institute for Chemical Carcinogenesis, Guangzhou Medical College, Guangzhou 510182, PR China

## Abstract

**Background:**

NBS1 is a key DNA repair protein in the homologous recombination repair pathway and a signal modifier in the intra-S phase checkpoint that plays important roles in maintaining genomic stability. The *NBS1 *8360G>C (*Glu185Gln*) is one of the most commonly studied polymorphisms of the gene for their association with risk of cancers, but the results are conflicting.

**Methods:**

We performed a meta-analysis using 16 eligible case-control studies (including 17 data sets) with a total of 9,734 patients and 10,325 controls to summarize the data on the association between the *NBS1 *8360G>C (E185Q) polymorphism and cancer risk.

**Results:**

Compared with the common 8360GG genotype, the carriers of variant genotypes (i.e., 8360 GC/CC) had a 1.06-fold elevated risk of cancer (95% CI = 1.00–1.12, *P *= 0.05) in a dominant genetic model as estimated in a fixed effect model. However, the association was not found in an additive genetic model (CC *vs *GG) (odds ratio, OR = 0.98, 95% CI = 0.85–1.13, *P *= 0.78) nor in a recessive genetic model (CC *vs *GC +GG) (OR = 0.94, 95% CI = 0.82–1.07, *P *= 0.36). The effect of the 8360G>C (E185Q) polymorphism was further evaluated in stratification analysis. It was demonstrated that the increased risk of cancer associated with 8360G>C variant genotypes was more pronounced in the Caucasians (OR = 1.07, 95% CI = 1.01–1.14, *P *= 0.03).

**Conclusion:**

Our meta-analysis suggests that the *NBS1 *E185Q variant genotypes (8360 *GC/CC*) might be associated with an increased risk of cancer, especially in Caucasians.

## Background

DNA damage may increase cancer risk, and DNA double-strand breaks (DSBs) cause the most potentially serious damage to the genome. If unrepaired, DSBs may lead to genomic instability and thus cancer [1]. The repair of DSBs in human cells includes two different pathways, homologous recombination repair (HR) and non-homologous end joining (NHEJ) pathways [2]. The initial step in both pathways is the recognition and signaling of DNA DSBs by a protein complex containing Nijmegen breakage syndrome 1 (NBS1), meiotic recombination 11 homologue (MRE11), and human RAD50 homologue (RAD50) proteins [3]. NBS1 plays an important role as a sensor in repairing the DSBs and activates the cell-cycle checkpoint signaling; it also directly binds to the phosphorylated histone H2AX that is located around DSBs, participating in maintaining genomic stability [4], and prevents cells from telomeric fusion [2,5]. A markedly impaired DSB repair was observed in cells from patients with Nijmegen breakage syndrome [6], in which cells are characteristic of chromosome instability and sensitivity to DSB-causative agents [7,8].

The *NBS1 *gene is located on human chromosome 8q21 and codes for a protein termed nibrin (754-amino acid protein) [9-11]. Some *NBS1 *mutations and polymorphisms have been reported to be associated with risk of several cancers, including cancers of the breast, lung, bladder, ovaries, non-Hodgkin lymphoma, malignant melanoma and basal cell carcinoma of the skin [12-18]. A homozygous 5-bp deletion in exon 6 (657del5) has been reported to be associated with an elevated risk of breast cancer in Polish and Russian populations [15,19]. However, the 657del5 variant appears to be a Slavic origin with a low frequency of approximate 0.5% in Eastern Europe populations and even lower in other ethnic groups. According to the Environmental Genome Project (EGP) SNP database of the (NIEHS) (http://egp.gs.washington.edu, accessed on March 1, 2008), 249 single nucleotide polymorphisms (SNPs) are reported, of which 84 are common polymorphisms (with a minor allele frequency > 5%). Among these SNPs, the 8360G>C (*Glu185Gln*, E185Q, rs1805794) is one of the most commonly studied polymorphisms. However, the results of studies on association between the 8360G>C (E185Q) polymorphism and the risk of cancers are conflicting [1,20,21]. To summarize the published data, we performed a meta-analysis from all eligible case-control studies to assess the association between the *NBS1 *E185Q polymorphism and cancer risk.

## Methods

### Bioinformatics Analysis

Based on the resequencing information about the *NBS1 *gene provided by the NIEHS Environmental Genome Project http://egp.gs.washington.edu/data/nbs1/, we calculated the D' value and *r*^2 ^coefficient by linkage disequilibrium (LD) analysis. In addition, we further analyzed SNPs in LD with 8360G>C in a 5-Mb region on chromosome 8 via the HapMap SNP database (release #36, http://www.hapmap.org/, March 1, 2008) that have genotypes for the CEPH trios with the ssSNPer web interface http://gump.qimr.edu.au/general/daleN/ssSNPer/[22].

### Literature search strategy for identification of the studies

We carried out a literature search in the PubMed and SciFinder Scholar (CA web version) database (between January 2000 and February 2008) to identify all articles that investigated the association between the *NBS1 *E185Q polymorphism and cancer risk in all ethnic groups, using the following keywords and subject terms: 'NBS1,' 'cancer,' and 'polymorphism.' We evaluated the titles and abstracts of all relevant publications first but excluded abstracts, case reports, editorials, and review articles. Studies included in the current meta-analysis had to meet the following criteria: (a) The study used a case-control study design; (b) the report described cancer diagnoses and the sources of cases and controls; (c) the report had available genotype frequency; (d) the authors offered the size of the samples, odds ratios (ORs) and their 95% confidence intervals (CIs); (e) the definition of the exposure or risk genotypes was similar in all reports; and (f) the methods of data collection and analysis were statistically acceptable.

### Data extraction

Data were collected on the *NBS1 *E185Q genotype for studies of different types of cancers. The first author, a year of publication, country, ethnicity of the study population, and the number of cases and controls and allele frequency were also described.

### Methods for quantitative synthesis

The Hardy-Weinberg equilibrium (*p*^2 ^+ 2*pq *+ *q*^2 ^= 1, where *p *is the frequency of the variant allele and *q *= 1 - *p*), was tested by goodness-of-fit Chi-square tests to compare the observed genotype frequencies with expected genotype frequencies in cancer-free controls for all studies. The selections of published studies used for meta-analysis were further evaluated in sensitivity analyses. Odds ratio (OR) and 95% confident interval (CI) in each case-control study was used to assess the strength of association between the *NBS1 *8360G>C (E185Q) genotypes and the risk of cancer in dominant (GC+CC *vs *GG), additive (CC *vs *GG), and recessive (CC *vs *GC +GG) genetic models. The combined OR was calculated according to the method of Woolf [23]. A *χ*^2^-based Q statistic test was performed to assess the between-study heterogeneity [24]. If the *P *value of heterogeneity test was ≥ 0.10, a fixed effect model using the Mantel-Haenszel method was used to calculate the combined OR, which assumed the same homogeneity of the effect size across all the studies. If the *P *value of the heterogeneity test was <0.10, it showed that the heterogeneity between-study was statistically significant. The random effects model using the DerSimonian and Laird method was performed to calculate the combined OR [25]. If there was no between-study heterogeneity, the results from those two methods calculating the combined OR would be identical. The significance of the combined OR was determined by the *Z*-test, in which *P*< 0.05 was considered significant. Finally, combined ORs and their 95% CIs were presented. Stratification analyses for different types of cancers were conducted for breast cancer, lung cancer, bladder cancer, basal cell carcinoma, and other cancers (i.e., ovarian, prostate, or colorectal cancer) to estimate cancer-specific OR. Stratification analyses by ethnicity were also conducted for Caucasian, Asian and African Americans populations to estimate ethnic-specific ORs.

Publication bias was assessed with the funnel plot, in which the standard error of log (OR) of each study was plotted against its OR value. An asymmetric plot suggested possible publication bias by the method of the Egger's linear regression test [26]. The significance of the intercept was determined by the Student *t*-test as suggested by Egger. If the *P*-value of Egger's linear regression test was less than 0.05, it meant that there was publication bias in the meta-analysis.

The SAS/Genetics software program (Version 9.1, SAS Institute, Inc., Cary, NC, USA) was used to determine the LD of SNP pairs and Hardy-Weinberg equilibrium. Other statistical software used included SPSS12.0 for windows software (SPSS Inc., Chicago, USA), software Stata version 7.0, and Review Manager (version 4.2, the Cochrane Collaboration). All *P*-values were two-sided.

## Results

### Literature search and meta-analysis databases

We found 31 epidemiologic studies using the search by '*NBS1*,' 'cancer' and 'polymorphism' through Pubmed and SciFinder Scholar (CA web version). Of these 31 studies, 15 studies were excluded, 9 studies were excluded because they were not case-control studies [27-35], 5 studies were excluded because E185Q polymorphism or its genotype frequency was not reported [14,36-39], and one studies focused on hematotoxicity but not cancer [40]. The remaining 16 case-control studies included 17 data sets (because Millian's study included two populations: African-American and whites) [1,13,16-18,20,21,41-49]. All the articles used DNA from blood samples for genotyping. We established a database for the extracted information from each article. Table 1 shows the essential information, including first author, cancer type, year of the publication, the numbers of cases and controls, and frequencies of *NSB1 *8360 C allele for all studies. There were six studies for breast cancer [1,20,21,41,42], three for the lung cancer [18,43,44], three for the bladder cancer [16,45,48], two for the basal cell carcinoma [17,46], one for the ovarian [13], one for prostate cancer [47], and one for colorectal cancer [49]. Among 17 eligible datasets included in the final analysis, there were 14 (82.3%) of Caucasians, two (11.8%) of Chinese, and one (5.9%) of African-Americans. Additional information is listed in the forest plots in our meta-analyses.

**Table 1:** Summary of eligible studies considered in the meta-analysis

First author(year)	Country	Ethnicity	Cancer type	Type of study	Case no.	Control no.	C Allele frequency (%) case/control
Kuschel(2002)[1]	Germany	Caucasians	Breast cancer	Population-based	1694	734	34.3/32.2
Forsti(2004)[20]	Finland	Caucasians	Breast cancer	Population-based	223	319	35.7/38.6
Millikan (2005)[41]	North Carolina	African-American	Breast cancer	Hospital-based	726	681	25.1/23.6
Millikan (2005)[41]	North Carolina	Caucasians	Breast cancer	Hospital-based	1273	1136	31.6/32.3
Lu(2006)[21]	USA	Caucasians	Breast cancer	Hospital-based	421	423	35.9/29.7
Zhang (2005)[42]	China	Chinese	Breast cancer	Hospital-based	220	310	35.9/38.2
Lan(2005)[18]	China	Chinese	Lung cancer	Population-based	118	111	57.2/66.7
Ryk (2006)[43]	Sweden	Caucasians	Lung cancer	Hospital-based	177	152	-/-
Zienoldding (2006)[44]	Norway and of Norwagian	Caucasians	Lung cancer	Hospital-based	376	310	34.4/28.5
Broberg (2005)[16]	Sweden	Caucasians	Bladder cancer	Hospital-based	61	154	36.1/37.3
Sanyal (2004)[45]	Sweden	Caucasians	Bladder cancer	Hospital-based	299	278	38.5/34.2
Figueroa(2007)[48]	Spanish	Caucasians	Bladder cancer	Hospital-based	1086	1020	31.8/30.0
Festa (2005)[17]	Sweden and Finland	Caucasians	Basal cell carcinoma	Hospital-based	241	574	37.1/36.8
Thirumaran (2006)[46]	Hungary, Romania, and Slovakia	Caucasians	Basal cell carcinoma	Hospital-based	529	533	35.6/32.4
Auranen (2005)[13]	Combined*	Caucasians	Ovarian cancer	Mixed^§^	1586	2685	32.7/33.3
Hebbring (2006)[47]	Finland	Caucasians	Prostate cancer	Population-based	200	200	35.5/35.8
Pardini (2008)[49]	Czech Republic	Caucasians	Colorectal cancer	Hospital-based	532	532	31.8/34.0

*: United Kingdom SEARCH study. Danish MALOVA study. United States FROC study. United Kingdom Royal Marsden Hospital and young ovarian cancer study (UK RMH/YOV).

^§^: Subjects including population and hospital source.

The frequency distributions of genotypes in control groups from all studies were in accordance with Hardy-Weinberg equilibrium (*P *> 0.05), except for Hebbring's study (*χ*^2 ^= 3.93, *P *= 0.05). We performed a sensitivity analysis to for the selection of published studies in the meta-analysis. The frequencies of the 8360 C allele in the control groups are also listed in Table 1. Compared with the reported frequency from the database of HapMap http://www.hapmap.org/cgi-perl/gbrowse/hapmap20_B35/, the frequencies of 8360 C allele in the meta-analysis were not as similar as that from the International HapMap Project. There might be population diversity. We would take that into account in the meta-analysis.

### Test for heterogeneity

**Figure 1:**
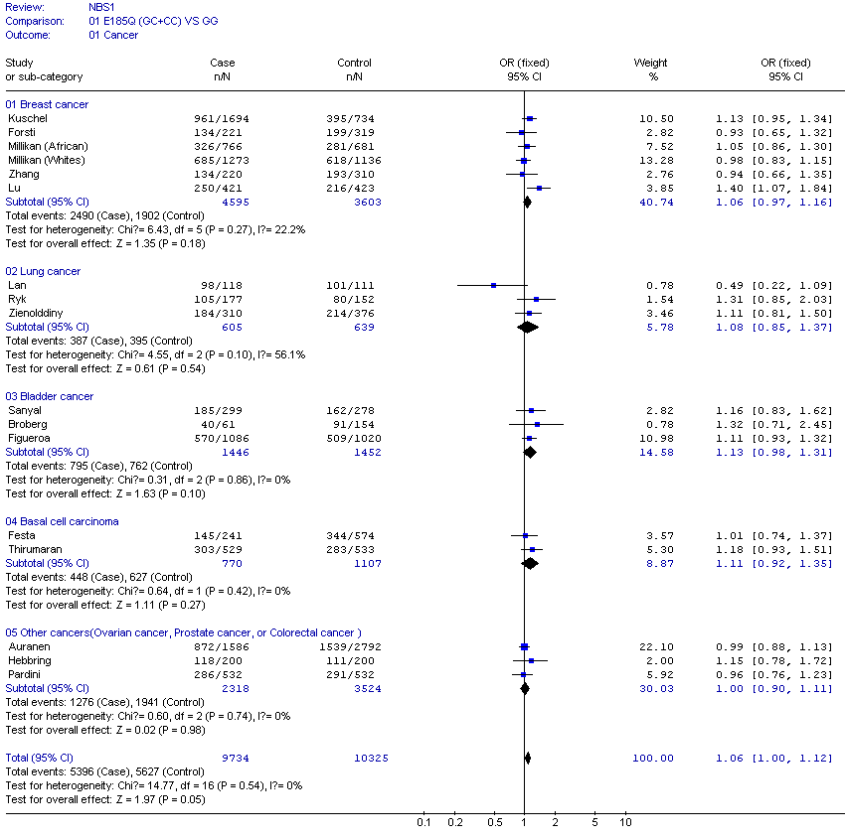
**Meta-analysis for NBS1 E185Q polymorphism variant genotypes (GC and CC) vs GG in different type of cancers (breast cancer, lung cancer, bladder cancer, basal cell carcinoma, ovarian cancer, prostate cancer, and colorectal cancer)**.

**Figure 2:**
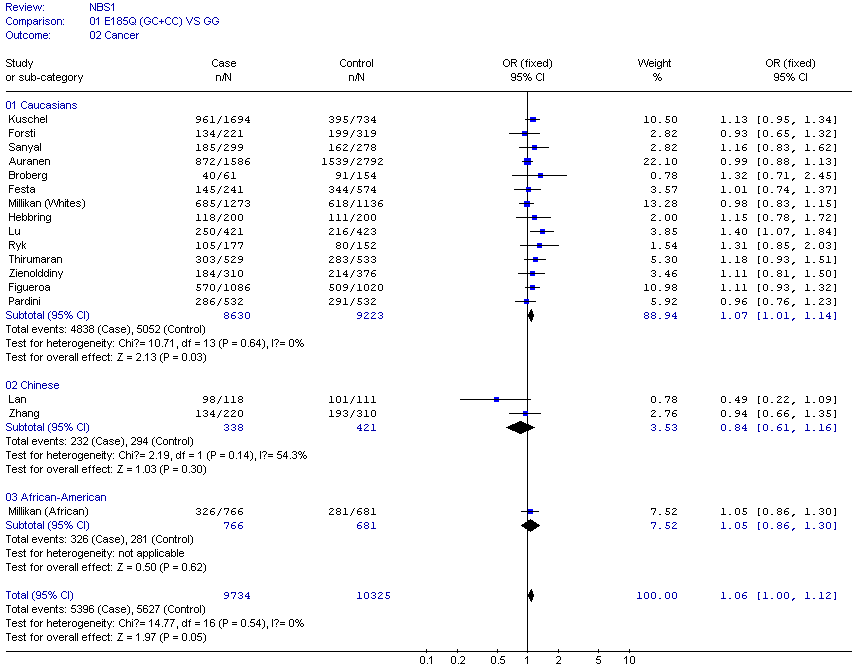
**Meta-analysis for NBS1 E185Q polymorphism variant genotypes (GC and CC) vs GG in different ethnicities (Caucasians, Chinese and African Americans)**.

Table 2 shows that no between-study heterogeneity was found in C *vs *G allele comparison for 16 datasets (*P *= 0.10) and in dominant genetic models for all 17 datasets (*P *= 0.54). However, there was between-study heterogeneity in the additive genetic model (*P *= 0.02) and recessive genetic model (*P *= 0.03) for 16 datasets. In the subgroup analyses by the type of cancers and ethnicity, as shown in Figures 1 and 2, the heterogeneity test did not show any significant difference in dominant genetic models: three lung cancer studies (*P *= 0.10), six breast cancer studies (*P *= 0.27), three bladder cancer studies (*P *= 0.86), two basal cell carcinoma studies (*P *= 0.42), other cancers (ovarian, prostate, and colorectal cancer) (*P *= 0.74), fourteen Caucasian population studies (*P *= 0.64), and two Chinese population studies (*P *= 0.14).

**Table 2:** Summary of ORs for various comparisons

E185Q comparison*	Population (number of data sets)^§^	Fixed-effects OR(95%CI)	Random-effects OR(95%CI)	Heterogeneity (*P *value Q test)	*P *value (fixed)	*P *value (random)
(GC+CC) *vs *GG	17	1.06(1.00–1.12)	1.06(1.00–1.12)	0.54	0.05	0.05
CC *vs *GG	16	0.99(0.90–1.09)	0.98(0.85–1.13)	0.02	0.83	0.78
CC *vs *(GC +GG)	16	0.95(0.87–1.04)	0.94(0.82–1.07)	0.03	0.28	0.36
C *vs *G	16	1.02(0.97–1.08)	1.02(0.98–1.07)	0.10	0.45	0.36

*Additive genetic model: CC *vs *GG, recessive genetic model: CC *vs *(GC +GG), dominant genetic model: (GC+CC) *vs *GG

^§^Ryk's study was not involved in 16 studies.

### Quantitative data synthesis

For the *NSB1 *E185Q polymorphism, the data available for our meta-analysis were obtained from 17 datasets consisted of 9,734 cases and 10,325 controls. Associations of the NBS1 E185Q allele and genotypes with cancer risk were estimated using dominant (GC+CC *vs *GG), additive (CC *vs *GG), and recessive (CC *vs *GC +GG) genetic models in either fixed or random effect models according the heterogeneity Q test in Table 2. There were 16 datasets in these comparisons, except for Ryk's study that only showed data in dominant genetic models. Compared with the wild-type 8360 GG genotype, the carriers of variant genotypes (i.e., *GC/CC*) had a 1.06-fold elevated risk of cancer (95% CI = 1.00–1.12, *P *= 0.05) as estimated in a fixed effect model for dominant genetic effects. We further performed a sensitivity analysis, and found that when Hebbring's study was excluded owing to the conflict of Hardy-Weinberg equilibrium, the combined ORs of cancer risk was still 1.06 (95% CI = 1.00–1.12), and the *P *value of the between-study heterogeneity test was decreased significantly (from *P *= 0.54 to *P *= 0.48). However, the association between the *NSB1 *E185Q polymorphism and cancer risk was not significant in the additive genetic model (CC *vs *GG) (OR = 0.98, 95% CI = 0.85–1.13, *P *= 0.78) nor in the recessive genetic model (CC *vs *GC + GG) (OR = 0.94, 95% CI = 0.82–1.07, *P *= 0.36).

The effect of 8360G>C (E185Q) polymorphism was further evaluated in stratification analysis. By the types of cancer, in those three lung cancer studies consisted of 605 cases and 639 controls, the variant genotypes (387 cases and 395 controls) had a non-significantly increased risk of lung cancer (OR = 1.08, 95% CI = 0.85–1.37, *P *= 0.54) as estimated in a fixed effect model (Figure 1). In the six breast cancer studies of 4,595 cases and 3,603 controls, the variant genotypes (2,490 cases and 1,902 controls) had a non-significantly increased risk (OR = 1.06, 95% CI = 0.97–1.16, *P *= 0.18) (Figure 1). Similarly, in the three bladder cancer studies of 1,446 cases and 1,452 controls (OR = 1.13, 95% CI = 0.98–1.31, *P *= 0.10) and three basal cell carcinoma studies of 770 cases and 1,107 controls (OR = 1.11, 95% CI = 0.92–1.35, *P *= 0.27) (Figure 1).

In the stratification analyses for ethnicity, we found that the increased risk of cancer associated with 8360G>C variant genotypes was more pronounced in the Caucasians (OR = 1.07, 95% CI = 1.01–1.14, *P *= 0.03), but not in Chinese (OR = 0.84, 95% CI = 0.61–1.16, *P *= 0.30) nor in African Americans (OR = 1.05, 95% CI = 0.86–1.30, *P *= 0.62) (Figure 2).

### Bias diagnostics

**Figure 3:**
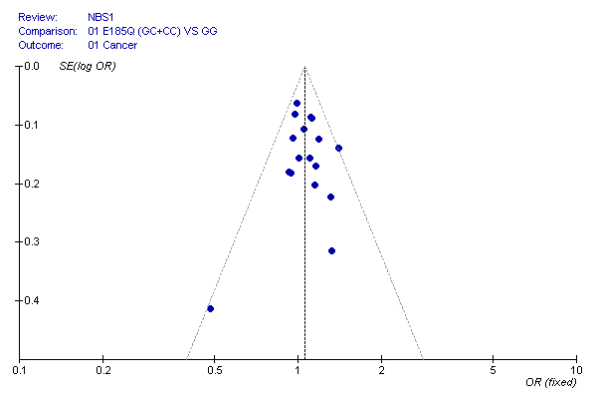
**Funnel plot of the Egger's test of NBS1E185Q polymorphism for publication bias**.

To evaluate publication biases, the *NSB1 *E185Q genotypes were plotted against the precision ones in a funnel plot, which is approximately symmetrical. Egger's test suggested that there was no publication bias in the current meta-analysis (*t *= 0.15, df = 16, *P *= 0.88). Furthermore, we found that the fail-safe number for the finding of *NSB1 *E185Q variant genotypes associated with 1.06 fold increased risk of cancer was 60, which suggests that biases from publications and other factors may not have a significant influence on the results of current meta-analysis for the association between *NSB1 *E185Q polymorphism and cancer risk (Figure 3).

## Discussion

In this meta-analysis consisted of 16 independent case-control studies with 17 datasets, we found that the carriers of *NBS1 *E185Q variant genotypes had a 1.06-fold increased risk of cancer in the dominant genetic model, and the association was more pronounced in the Caucasians. However, we did not find evidence for significant associations in the subgroup analysis for the individual type of cancers, such as lung cancer, breast cancer, basal cell carcinoma, urogenital cancers (i.e., bladder cancer, ovarian cancer, and prostate cancer), or colorectal cancer.

The *NBS1 *8360G>C (rs1805794) polymorphism is a non-synonymous SNP with an amino acid change (Glu185>Gln or E185Q). Either one or two missense changes in the dominant genetic model may affect the function of NBS1 and interfere protein-protein interaction [50]. The E185Q amino acid substitution was predicted to be tolerated via the SIFT prediction tool http://blocks.fhcrc.org/sift/SIFT.html. The E185Q SNP is located in a breast cancer carboxy-terminal (BRCT) domain (108–196 amino acids) of *NBS1 *[3], and such a domain facilitates NBS1 to interact with BRCA1 (one of two familial breast cancers mutated genes) forming BRCA1-associated genome surveillance complex (BASC), which is responsible for the recognition and repair of aberrant DNA [51].

In the LD analysis, we found that the E185Q SNP was in a completed LD (D' = 1.00, *r*^2 ^= 1.00) with the loci 626G>A in the promoter of *NBS1 *(-1418 nt to initiation transcription code ATG), 3816G>A (*Leu34Leu*, rs1063045) and 40419A>G (*Pro672Pro*, rs1061302) [21]. In further bioinformatics analysis searching for the transcription factor binding sites with the TFSEARCH program http://mbs.cbrc.jp/research/db/TFSEARCH.html, we found that the 626G allele, but not the 626A allele, creates a new binding site of SRY that is a functional target of the tumor suppression gene *WT1 *[52], and that the 626G allele in LD with Glu185 may function in prohibiting carcinogenesis. Furthermore, in a region from 35309 bp upstream to 29477 bp downstream of E185Q on chromosome 8, we found that there were thirty-three polymorphisms being in completely LD (all with an *r*^2 ^= 1.00) with E185Q (data not shown). Because the exact molecular mechanism involving the *NBS1 *E185Q variant in the etiology of cancer is still unclear, further investigations are needed to identify its LD with other unknown functional variants of cancer susceptibility candidate genes.

DNA DSBs in human cells is one of the major factors for carcinogenesis. Affected individuals when exposed to different carcinogens will have different outcomes. For example, the common *NBS1 *185Gln allele has been associated with an increased risk for lung cancer in a Chinese population exposed to smoky coal emissions [18]. Stable covalent BaP DNA adducts can cause single-strand breaks, resulting in DSBs during replication [53]. The established risk factors for bladder cancer include cigarette smoking, exposure to industrially related aromatic amines and drugs [54,55]. Some common, low-penetrance susceptibility genes in human populations may interact with radiation exposure to increase risk of breast cancer [56], because DNA DSBs are frequently induced by ionizing radiation, which may result in altered apoptosis or tumorigenesis [1]. For ovarian cancer, however, ovulation may play a role in ovarian cancer development [13]. Smoking and drinking habit are frequently associated with colorectal cancer risk [57]. BCCs are caused by interplay between genetic and environment factors, too [17]. The *NBS1 *E185Q variant genotypes (8360*GC/CC*) were found to be associated with a *p53 *mutation in lung cancer, suggesting a role in lung carcinogenesis [32]. Other report suggested that the *XRCC3 *interacted with *NBS1 *involved in the homologous recombination [58]. Therefore, it is likely that the *NBS1 *E185Q polymorphism may modify cancer susceptibility via gene-environment and gene-gene interactions. However, not all studies offer the information about environmental exposure.

Sensitivity analysis showed that for breast cancer no association (combined OR = 1.05, 95% CI = 0.99–1.11) was present after exclusion of the study of Lu. In Lu's study the subjects were ≤ 55 old women [21]. About 40% of NBS patients develop cancer before the age of 21 [59]. It has been suggested that there are different etiologic pathways for early-onset and late-onset types of breast cancer [60,61]. Therefore, large studies of *NBS1 *E185Q stratified by age are needed to investigate the inter-individual susceptibility to cancer.

There appeared to be ethnicity-specific genetic effects, because we found that the association between 8360G>C variant genotypes and increased risk of cancer was significant only in Caucasians but not in Chinese or African-Americans, suggesting genetic diversity among different ethnicities. The frequencies of 8360 C allele in the controls of selected studies were not as similar as that from the database of the International HapMap Project. We would take that into account when applying to the findings from the meta-analysis.

Potential publication bias may exist in this meta-analysis, because the studies with negative results are more likely not to be published, though there was no testable publication bias in our meta-analysis by the funnel plot. Because there were only four out of 17 datasets were population-based case-control studies, others being hospital-based case-control studies, the study subjects may not be representative of the general population and could lead to selection bias.

## Conclusion

In conclusion, our meta-analysis suggests that based on the published data, the *NBS1 *E185Q variant genotypes (8360 *GC/CC*) might be associated with an increased risk of cancer, especially in Caucasians. Due to the limitations of such meta-analysis, larger association studies or multicentric case-control studies and the studies assessing gene-environment interactions are warranted to confirm these findings.

## Abbreviations

**NBS**: Nijimegen break syndrome; ***NBS1***: Nijimegen break syndrome mutated gene; **DSBs**: DNA double strand breaks; **SNP**: single nucleotide polymorphism; **OR**: odds ratio; **CI**: confidence interval.

## Competing interests

The authors declare that they have no competing interests.

## Authors' contributions

ML participated in study design and drafted the manuscript. JL carried out Bioinformatics Analysis and critically revised the manuscript. XY, MY, and HT performed the statistical analysis and participated in the critical revision of the manuscript. BY participated in collection of data and manuscript preparation. LS participated in its design. All authors read and approved the final manuscript.

## Pre-publication history

The pre-publication history for this paper can be accessed here:

http://www.biomedcentral.com/1471-2407/9/124/prepub
